# Do morphogenetic switching and intraspecies variation enhance virulence of *Candida auris*?

**DOI:** 10.1371/journal.ppat.1012559

**Published:** 2024-10-15

**Authors:** Trinh Phan-Canh, Karl Kuchler

**Affiliations:** 1 Max Perutz Labs Vienna, Vienna Biocenter Campus (VBC), Vienna, Austria; 2 Center for Medical Biochemistry, Medical University of Vienna, Vienna, Austria; Brown University, UNITED STATES OF AMERICA

## Abstract

Intraspecies variations that affect pathogenicity and antifungal resistance traits pose a serious obstacle to efficient therapy of *Candida auris* infections. Recent reports indicate that mutations determine drug susceptibility and virulence. However, mutations alone cannot fully explain a bewildering variety of phenotypes in clinical isolates from known *C*. *auris* clades, suggesting an unprecedented complexity underlying virulence traits and antifungal resistance. Hence, we wish to discuss how phenotypic plasticity promotes morphogenetic switching and how that contributes to intraspecies variations in the human fungal pathogen *C*. *auris*. Further, we will also discuss how intraspecies variations and morphogenetic events can impact the progress in molecular mycology research that aims to find better treatments for *C*. *auris* infections. Finally, we will present our opinion as to the most relevant questions to be addressed when trying to better understand the pathophysiology of *C*. *auris*.

## Introduction

The wide variations of ambient temperatures and additional environmental challenges such as global overuse of agrochemicals without proper sewage treatment [1,2] cause ecological imbalances. This may facilitate evolutionary changes and thus establish potential pathogenesis traits. The increase in the amplitude and frequency of extreme events such as very hot or cold days, storms with flooding, or drought may promote the genetic and epigenetic selection of environmental pathogens with altered pathophysiology, including a switch in host range, as well as the rise of anti-infective drug resistance. Moreover, thawing of permafrost regions may even liberate prehistoric pathogens of hitherto unknown medical impact [3]. Hence, newly emerging pathogens could pose unprecedented healthcare threats to humanity.

Notably, the growing incidence of age-related immunological diseases, prevalent anticancer therapy, transplantation, or longevity has been promoting life-threatening invasive fungal diseases. Among an estimated 3 million fungi in our environment, only a few dozen species cause about 6.5 million invasive infections, claiming some 3.8 million annual deaths globally [4]. Recognizing these threats, the World Health Organization (WHO) has, for the first time, defined a priority list of fungal pathogens requiring immediate attention in both research and antifungal drug discovery [5]. This list includes *Candida auris*, an emerging pathogen exhibiting remarkable skin tropism and pan-antifungal resistance traits [6]. *C*. *auris* has been thriving in wetlands, niches that often constitute severe stress environments due to the accumulation of agrochemicals and salinization, which is further exacerbated by global warming [7–9]. However, *C*. *auris* has spread globally, causing high-mortality outbreaks of systemic infections in hospitals and nursing homes [10–12]. Accelerating global warming promoted *C*. *auris* to alter its environmental niche as well as host range, suggesting a causal link between climate change and the human colonization by *C*. *auris* [13]. Most likely, the six known *C*. *auris* clades emerged from independent but convergent evolutionary events [7,8,14,15]. Importantly, *C*. *auris* is a critical human fungal pathogen that can acquire clinical resistance to all antifungal drugs in use [16–19], implying that environmental stress can impact resistance development [20–22]. Additional examples for the impact of climate change on fungal pathogens include *Trichophyton indotineae* [23], which displays significant terbinafine resistance [24], and *Rhodosporidiobolus fluvialis*, developing pan-resistance when facing mammalian body temperature [21].

Moreover, heat stress can induce genomic rearrangements by affecting transposon mobility [25] and/or changes in mutation rates [21], thereby altering drug resistance and virulence in fungi. Indeed, *C*. *auris* undergoes rapid evolution in systemic infections, yielding aggregated cells, which carry de novo point mutations affecting virulence as well as antifungal resistance through biofilm formation [26]. However, intraclade genetic variations are low and mainly found in noncoding regions [27,28]. Therefore, we speculate that genomic rearrangements and mutations in noncoding regions or multistable epigenetic changes affect intraspecies variations of *C*. *auris*.

## Multistable cell fate switching determines pathogenic traits under environmental stress

**Fig 1:**
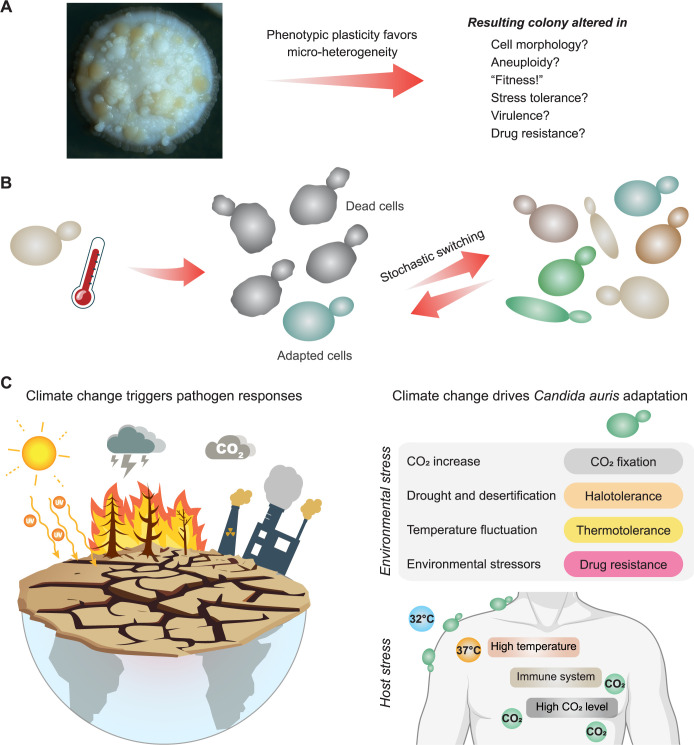
Cell-fate switching and morphogenesis in *C*. *auris* may be linked to pathogenicity. (**A**) Patient isolates encountering host defense may undergo stochastic switching events to yield pools of multi-stable cell types. (**B**) Switching creates a population of microheterogeneic cells with distinct fitness and growth properties. (**C**) Climate change shapes stress resistance and pathogenic traits of *C*. *auris*. Figures use modified drawings sourced from Openclipart and an Adobe Illustrator’s AI tool. The human body illustration in Fig 1C was created with BioRender.

In addition to its extreme skin tropism, *C*. *auris* displays another as yet underexplored trait that appears linked to the adaptation in different environments and host niches [26,29,30]. For example, haploid *C*. *auris* undergoes morphotype switching, which may be related to the heritable epigenetic *White-Opaque* switch in the obligatory diploid *Candida albicans* [31–34]. While *C*. *auris* does not require mating competence, high temperatures do promote switching (Fig 1A and 1B) [7,28]. This may reflect unprecedented complexities and dynamics of intraspecies strain-to-strain variations in this fungus [35]. Of note, the immune surveillance that *C*. *auris* faces on the skin surface microenvironment and systemic infection may provide further switching triggers [26,29]. Interestingly, the distantly related fruit yeast *Torulaspora microellipsoides* shows similar phenomena of morphogenesis [36], supporting the notion that morphogenetic switching may be a key driver of adaptive evolution when fungi encounter environmental threats, fluctuating growth conditions, nutrient limitation [37], or immune defense [21,26].

We recently screened morphotypes of more than 100 *C*. *auris* clinical isolates representing all clades. Strikingly, we discovered a broad spectrum of colony pigmentation as well as numerous distinct morphotypes (Fig 1A). Although *C*. *auris* is thought to be thermotolerant [35], some isolates are in fact temperature-sensitive at 42°C [9,38]. The evolutionary pace is remarkable, as adapted colonies emerge after only 3 to 4 days, suggesting adaptation takes a mere of 25 to 30 generation times. Switching most likely integrates extensive epigenetic mechanisms with transcriptional rewiring of networks, possibly including temporary ploidy alterations [39] as in *Torulaspora microellipsoides* [36]. Adapted colonies exhibit a range of morphologies and phenotypes, including altered fitness and pigmentation (Fig 1A). This is manifested by a gradient of brownish colony colors, literally yielding “fifty shades of *Brown*” patterns (Fig 1A). Hence, we refer to it as *White-Brown* (W/B) morphogenetic switching, although we anticipate that additional hitherto unrecognized cell-fate decision mechanisms may exist (Fig 1B).

While *White-Brown* switching has not been reported in *C*. *auris*, some clinical isolates can form *White* and *Pink* colonies on Chromagar or Phloxine B agar, which also seems to be reversible [40,41]. Interestingly, *Pink* and *White* morphologies appear distinct regarding their ability for hyphal growth, aggregation, and biofilm formation [40–42]. This implies additional specific functions of W/B conversion in establishing or maintaining skin tropism, immune evasion, and virulence [26,40,42]. The W/B conversion creates a pool of microheterogeneic *C*. *auris* cell populations (Fig 1A), where individual cellular morphotypes can acquire various traits, perhaps depending on the tissue microenvironment or the quality of immune surveillance. Noteworthy, filamentation as well as biofilm formation are additional traits associated with pathogenicity and antifungal resistance of many fungal pathogens [21,43]. Indeed, the extreme skin adherence of *C*. *auris* facilitates biofilm formation [42] engaging pseudohyphal-like morphologies, especially when facing host immune defense in deeper skin layers [30,40].

The molecular mechanisms and complex dynamics behind W/B switching in *C*. *auris* remain to be explored. *C*. *auris* may employ a bet-hedging strategy to cope with environmental or host challenges, similar to other microorganisms [44]. Typically, regulatory feedback loops maintain bistable states, where two subpopulations form an equilibrium with overlapping but distinct expression profiles in a given ecosystem. An example are fungal or bacterial *persister* cells that emerge during antimicrobial treatment [45]. Similarly, *Salmonella* spp. can generate fast- and slow-dividing subpopulations when facing macrophage attacks, thus illustrating how cell-fate switching provides critical adaptive benefits for microbes encountering immune defense [46]. Remarkably though, *C*. *auris*, as well as some other microbial pathogens [47], appear capable of establishing multistable yet reversible phenotypic states (Fig 1A and 1B), suggesting the involvement of regulatory networks that contribute to the adaptive evolution of virulence. Since this may occur independently or in addition to mutational variations [26,42], *C*. *auris* is able to rely upon extreme phenotypic plasticity (Fig 1A and 1B). Although transcriptional profiles for W/O of *C*. *albicans* and *T*. *microellipsoides* are related, the underlying regulatory networks are distinct [36]. Interestingly enough, *C*. *auris* and *T*. *microellipsoides* share apples as an environmental niche, suggesting that both fungi face similar stressors during their life cycles, including fungicides and pesticides used for crop protection [48]. Indeed, crop protection chemicals are perhaps among the most prominent environmental drivers of drug resistance traits [1].

The impact of climate change is seen on a daily basis, including increasing levels of the atmospheric greenhouse gas CO_2_ posing severe stress to microorganisms (Fig 1C). Strikingly, we recently discovered that *C*. *auris* growing on human skin can fix minute levels of CO_2_ from ambient air so as to cope with nutrient limitation and to sustain active energy metabolism (available as a bioRxiv preprint) [49]. Moreover, most clinical *C*. *auris* isolates are both halo- and thermotolerant, traits that were necessary for the changes in the host range from environmental niches to humans. Notably, environmental *C*. *auris* strains grow slower at high temperature than clinical strains, implying rapid adaptive changes in the host [9] as observed under *in vitro* growth conditions. Hence, it seems reasonable to speculate that the higher human body temperature in deeper skin layers, along with increasing immune defense, are key parameters driving such morphogenetic complexity linked to drug resistance [21]. Mechanistically, *C*. *auris* perhaps exploits the phenotypic plasticity to maintain multiple cell states, thereby enhancing its ability to thrive in diverse niches (Fig 1B and 1C). In fact, it is tantalizing to speculate that such multistable cell states are reminiscent of stem cells that develop into distinct populations depending on stress signals or environmental cues. Remarkably, multiple cell-fate switching has also been reported for *T*. *microellipsoides* [36], thus highlighting the role of rapid evolution for intraspecies variations that affect virulence and resistance traits under climate change.

## Burning questions

However, despite intense global efforts, burning questions about *C*. *auris* pathophysiology remain unanswered [50]. As discussed here, multistable *White-Brown* switching can generate subpopulations of cells with distinct fitness levels as well as sensitivity for morphogenetic changes. That may involve rewiring of genetic regulatory networks, including those controlling metabolism or antifungal susceptibility to support skin tropism. This notion leads to the first question: How do extreme environmental temperatures and mammalian heat define intraspecies variations linked to distinct virulence traits evolving in individual hosts? It seems that clinical therapy will have to deal with *“patient-specific personalized”* virulence and drug susceptibility traits of *C*. *auris*. This makes efficient therapy a serious challenge but also poses severe impediments to molecular medical mycology research at large, especially when comparing datasets from different patient isolates. Although the Centers for Disease Control and Prevention (CDC) established a panel of around 20 *C*. *auris* isolates, a database or standard guidelines for benchmarking phenotypic data for clinical isolates from different geographical regions are still lacking. Such a database could significantly support research efforts by enabling the selection of reference strains that accurately represent the predominant traits of the species. Hence, it will help to reduce duplication of efforts and support the interpretation of (conflicting or inconsistent) data from different laboratories and, at the end, boost the progress aimed to find new therapies.

Second, owing to the complexity of phenotypic plasticity, the most important need is to address what are the regulatory networks that control the equilibrium of *White-Brown* conversion or the stability of *White* versus *Brown* cells in a given microenvironment? How can distinct multidrug resistance traits emerge in *White* or *Brown* morphotypes without showing an impact on fitness?

Third, how does skin colonization impact *White-Brown* conversion and to what extent do other immune defense mechanisms trigger morphogenesis or even immune evasion? Further, how can *C*. *auris* maintain efficient skin colonization where nutrients as well as trace elements are of low abundance? *C*. *auris* may rely on alternative metabolic strategies that allow for sustaining basal metabolism by scavenging nutrients from the skin and by communicating with skin microbiota that produce and release metabolic products such as short-chain fatty acids and carbon dioxide [49]. For example, the ability to metabolize minute amounts of carbon dioxide from ambient air may allow *C*. *auris* to maintain growth on skin without the need for invasion into deeper skin tissues [49]. Is this the main explanation for the efficient immune evasion or are the pronounced adhesive traits preventing pathogen clearing? Of note, *C*. *auris* releases proteases and it can form (pseudo)-filaments [29,30,35,40,50], yet in sharp contrast to pleiomorphic *C*. *albicans*, it is unable to efficiently breach intact epithelial barriers such as present in healthy skin [30].

Finally, one of the most tantalizing questions is why and how *C*. *auris* can acquire extensive phenotypic variations in clinical isolates from individual patients [35]. Are intraspecies variations resulting from genetic and epigenetic events or both cooperating through additive, synergistic or antagonistic mechanisms? Another critical point is how these factors affect the growth behavior of clinical isolates *in vitro* after being collected from patients and cultivated *in vitro* for many generations. Answering these questions will not only provide deeper mechanistic insights into fungal evolutionary processes that can shape pathogenesis and virulence of *C*. *auris* but also help to develop better antifungal therapy regimens at large.
